# Comparison of serum inflammatory indicators and radiographic results in MAKO robotic-assisted versus conventional total knee arthroplasty for knee osteoarthritis: a retrospective study of Chinese patients

**DOI:** 10.1186/s12891-022-05373-y

**Published:** 2022-05-04

**Authors:** Jia-Zheng Xu, Liang-Liang Li, Jun Fu, Chi Xu, Guo-Qiang Zhang, Wei Chai, Li-Bo Hao, Xiang Li, Ji-Ying Chen

**Affiliations:** 1 Senior Department of Orthopaedics, the Fourth Medical Center of Chinese PLA General Hospital, Beijing, 100142 China; 2 Department of Orthopaedics, The First Medical Center, Chinese PLA General Hospital, No. 28 Fuxing Road, Haidian District, Beijing, 100853 China; 3 National Clinical Research Center for Orthopaedics, Sports Medicine & Rehabilitation, Beijing, 100853 China; 4 Department of Orthopaedics, The Second Hospital of Shanxi Medical University, Taiyuan, Shanxi China

**Keywords:** Robot-assisted, Total knee arthroplasty, Prosthesis position, Early outcome, Inflammatory markers

## Abstract

**Background:**

The purpose of this study was to compare the serum inflammatory indicators and radiographic results of conventional manual total knee arthroplasty (CM-TKA) with those of MAKO-robotic assisted total knee arthroplasty (MA-TKA).

**Methods:**

We retrospectively analysed 65 patients with knee osteoarthritis who underwent unilateral TKA from December 2020 to November 2021 in our department, which included 34 patients who underwent MA-TKA and 31 patients who underwent CM-TKA. The tourniquet time and estimated blood loss (EBL) were compared between the two groups. Knee function was evaluated using range of motion (ROM), functional score and pain score. Leukocytes, C-reactive protein (CRP), erythrocyte sedimentation rate (ESR), interleukin-6 (IL-6), creatine kinase (CK), and neutrophil-to-lymphocyte ratio (NLR) were recorded at 3 time points (preoperative, and on the first and third postoperative days). The hip-knee-ankle angle (HKA) and the femoral and tibial component angles in the coronal and sagittal planes were used for postoperative radiographic evaluation.

**Results:**

The postoperative MA-TKA group had less EBL (496.9 ± 257.8 vs. 773.0 ± 301.3 ml, *p* < 0.001). There was no significant difference in knee function scores at 6 weeks postoperatively (*p* > 0.05). IL-6 levels were significantly lower in the MA-TKA group on the 1st postoperative day (11.4 (5.2, 21.0) vs. 24.6 (86.3, 170.8), *p* = 0.031). This difference in inflammatory indices became more pronounced at 72 hours after the operation because CRP, ESR, IL-6, and CK values were significantly lower in the MA-TKA group on the 3rd postoperative day (72 h) (*p* < 0.05). Postoperative radiographic examinations performed 2 days after the MA-TKA group suggested that only 2 cases of HKA had outlier values, which was remarkably better than the 12 cases found in the CM-TKA group (5.9% vs. 38.7%, *p* < 0.001). The frontal femoral component was significantly closer to the expected value of 90° in the MA-TKA group (90.9 (90.5, 92.3) vs. 92.4 (91.3, 93.7), *p* = 0.031). The remaining imaging evaluation parameters were not significantly different between the two groups (*p* > 0.05).

**Conclusions:**

In Chinese patients with OA, there was a milder systemic inflammatory response in the early postoperative period after MA-TKA compared to that of CM-TKA, as well as better radiographic outcomes. However, the tourniquet time was prolonged, and no advantages were observed in terms of functional score or pain score in the short-term follow-up.

## Background

As total knee arthroplasty technology advances, robot-assisted total knee arthroplasty (RA-TKA) is increasingly used. The MAKO surgical robot (Stryker, USA) is currently the most well-known joint replacement-assisting robot and one of the most widely used orthopaedic joint replacement robots in several countries. Whether robotic assistance has a more positive impact on early functional recovery after TKA remains controversial. RA-TKA has been widely reported to improve precision, provide better prosthesis position and lower extremity force lines, and obtain better imaging and functional outcomes than conventional surgery [1–3]. However, some studies have also concluded that there is no major difference in postoperative function and pain scores between robot-assisted and conventional manual total knee arthroplasty (CM-TKA) [4–6]. F. S. Haddad et al. analysed the inflammatory indicators after RA-TKA and CM-TKA and found that there was a transient mild systemic inflammatory response during the early postoperative period (Day 7) after RA-TKA [7]. The study evaluated the effect of different surgical procedures on the surgical site by analysing serum markers. We consider that the difference in inflammatory indicators caused by different surgical approaches in postoperative patients is underestimated. However, the peak change in C-reactive protein (CRP) in the trajectory of inflammatory indices after TKA was at 72 h, while interleukin-6 (IL-6) was at 48-72 h [8]. Therefore, for some indicators, 72 hours (before and after the peak) may be a more representative and significantly different time point, which was not mentioned in the study of Haddad et al.

Gandhi et al. found that Asian patients presented for surgery at a younger age and had a lower mean BMI in addition to greater pain and dysfunction than their white counterparts [9]. Due to the heterogeneity of the population and regional differences [10], patients undergoing an initial TKA in China often have more severe knee osteoarthritis. Ke Li et al. showed that Chinese knees were more trapezoidal and asymmetric with shallower trochleae [11]. Thus, the clinical outcomes of robot-assisted TKA in Western countries, as described in previous studies, may not be fully applicable to the Chinese population. There are no studies on the early clinical outcomes of MAKO robot-assisted TKA (MA-TKA) in the Chinese population. The purpose of this study was to (i) investigate the differences in inflammatory indicators and changes in muscle damage markers at 72 h after MA-TKA and CM-TKA and (2) investigate whether there are differences in joint function, pain scores and imaging outcomes in the early postoperative period between MA-TKA and CM-TKA in the Chinese population. The research hypothesis was that there would be a significant difference in serum inflammatory indicators between the two groups at 72 h postoperatively and that patients in the MA-TKA group would have better radiographic results and early functional scores.

## Methods

### Inclusion/exclusion criteria

We initially performed MA-TKA in May 2021 and 119 operations have been performed to date. Inclusion criteria consisted of the following: Patients who failed to respond to conservative treatment and underwent unilateral TKA for end-stage KOA (knee K-L classification of grade III or IV) between December 2020 and November 2021 were included. Exclusion criteria consisted of the following: (1) patients who underwent bilateral simultaneous TKA, (2) patients with neuromuscular dysfunction that affected lower limb function, (3) patients with severe coagulation dysfunction, (4) patients with haemoglobin < 80 g/L, which indicates severe anaemia, (5) patients with serious medical or surgical diseases or weaknesses that cannot tolerate surgery, (6) patients with rheumatoid arthritis and other autoimmune diseases, and (7) patients with a history of open knee surgery.

A total of 65 patients were included in this study according to the abovementioned nadir criteria, including 34 in the MA-TKA group and 31 in the CM-TKA group.

### General information

The MA-TKA group included the following participant characteristics: 14 left knees and 20 right knees of 5 males and 29 females aged 54-79 years, with a mean of 65.7 years; a body mass index (BMI) of 27.2 ± 2.5 kg/m^2^. The CM-TKA group included the following participant characteristics: 17 left knees and 20 right knees of 5 males and 26 females aged 54-83 years, with a mean of 68.5 years; a BMI of 27.9 ± 3.4 kg/m^2^.

The general data of age, sex, BMI, surgical side, preoperative knee K-L classification and hip-knee-ankle angle (HKA) deformity were not significantly different between the two groups (*P* > 0.05) and were thus comparable (Table 1).

**Table 1:** Demographic data and preoperative radiographic aberrations in the RA-TKA and CM-TKA groups

Parameter	MA-TKA (*n* = 34)	CM-TKA (*n* = 31)	*P* value
**Age (years)**	65.7 ± 6.7	68.5 ± 9.6	0.175
**Number of males/females (n,%)**			0.874
Males	5 (14.7)	5 (16.1)	
Females	29 (85.3)	26 (83.9)	
**BMI (kg/m**^**2**^**)**	27.2 ± 2.5	27.9 ± 3.4	0.327
**Operative side (n,%)**			0.271
Left	14 (41.2)	17 (54.8)	
Right	20 (58.8)	14 (45.2)	
**K-L grade (n, %)**			0.804
III	10 (29.4)	10 (32.2)	
IV	24 (70.6)	21 (67.8)	
**HKA angle(°)**	174.7 ± 3.1	173.5 ± 4.3	0.183

*MA-TKA* Mako-robotic assisted total knee arthroplasty, *CM-TKA* conventional manual total knee arthroplasty, *BMI* body mass index, *HKA* hip-knee-ankle, *K-L grade* Kellgren-Lawrence grade

### Surgical method

All operations were performed by 2 surgeons experienced in joint replacement. The surgeons all passed through their learning curve (performing more than 20 MA-TKAs of other types and a large number of MAKO robot-assisted THAs). A tourniquet was applied (inflated from the cutaneous tourniquet until the bone cement set and was released). An anteromedial incision for a medial parapatellar approach was made. Both groups used a fixed platform, Triathlon PS prosthesis (Stryker, USA), and were cemented.

The MA-TKA group was planned preoperatively, and the MAKO navigation system (Mako Robotic Arm Interactive Orthopaedic System) was used to assess anatomic landmarks based on preoperative CT 3D reconstruction. Important bony landmarks were appropriately shown intraoperatively. Femoral and tibial pins were placed at the superior border of the patella and below the tibial tuberosity, respectively, and a tracker was attached to the pins for optical motion capture and tracking. The physician then used the probe to “register” the specified anatomic position, and the system evaluated the knee soft tissue tension and flexion-extension gap in real time once the registration was completed. When the system indicated that automatic alignment was complete, the osteotomy began. The amount of bone to be removed is shown in green in the CT reconstructed lower limb model. When the green portion of the bone to be osteotomized was completely cleared in the navigation view, the osteotomy was completed on the current osteotomy surface. Further capsular or ligamentous release was performed when needed. After completion of the osteotomy, a trial mould of the prosthesis was installed, knee mobility and stability were assessed, the prosthesis was installed, the incision was sutured, and the femoral and tibial tracers and fixation nails were removed.

In the CM-TKA group, osteotomy of the proximal tibia was performed using the extramedullary localization method after skinning, followed by osteotomy of the distal femur using the intramedullary localization method.

### Rehabilitation treatment

Patients in both groups received the same treatment, which included an intravenous injection and topical infusion of tranexamic acid [12]. Postoperatively, both groups adopted the clinical pathway of Enhanced Recovery After Surgery (ERAS) [13]. Anti-infectives, analgesics and anticoagulants were administered to patients in both groups. Weight-bearing walking exercises with a floor walker in addition to nonweight-bearing knee flexion and extension exercises [14, 15] were recommended according to the patient’s recovery status. Complications, such as surgical site infection and vascular nerve injury, were screened throughout the procedure and therapy.

### Efficacy evaluation index

#### Clinical outcome evaluation

The operating time and intraoperative tourniquet application time were recorded for both groups; the estimated blood loss (EBL) was calculated based on the patient’s height and weight and the preoperative and postoperative 72-hour haemoglobin changes [16]; and range of motion (ROM), knee society score (KSS), and The Western Ontario McMaster Universities Osteoarthritis (WOMAC) Index were recorded preoperatively and at 6 weeks postoperatively to evaluate knee function and pain.

### Evaluation of serum inflammatory indices

Leukocytes, neutrophils, lymphocytes, C-reactive protein (CRP), erythrocyte sedimentation rate (ESR), interleukin-6 (IL-6), creatine kinase (CK) and neutrophil-to-lymphocyte ratio (NLR) were recorded preoperatively, the day after surgery and 3 days after surgery, to compare the differences in postoperative inflammatory indices and muscle damage indices between the two groups.

### Radiographic evaluation metrics

**Fig. 1:**
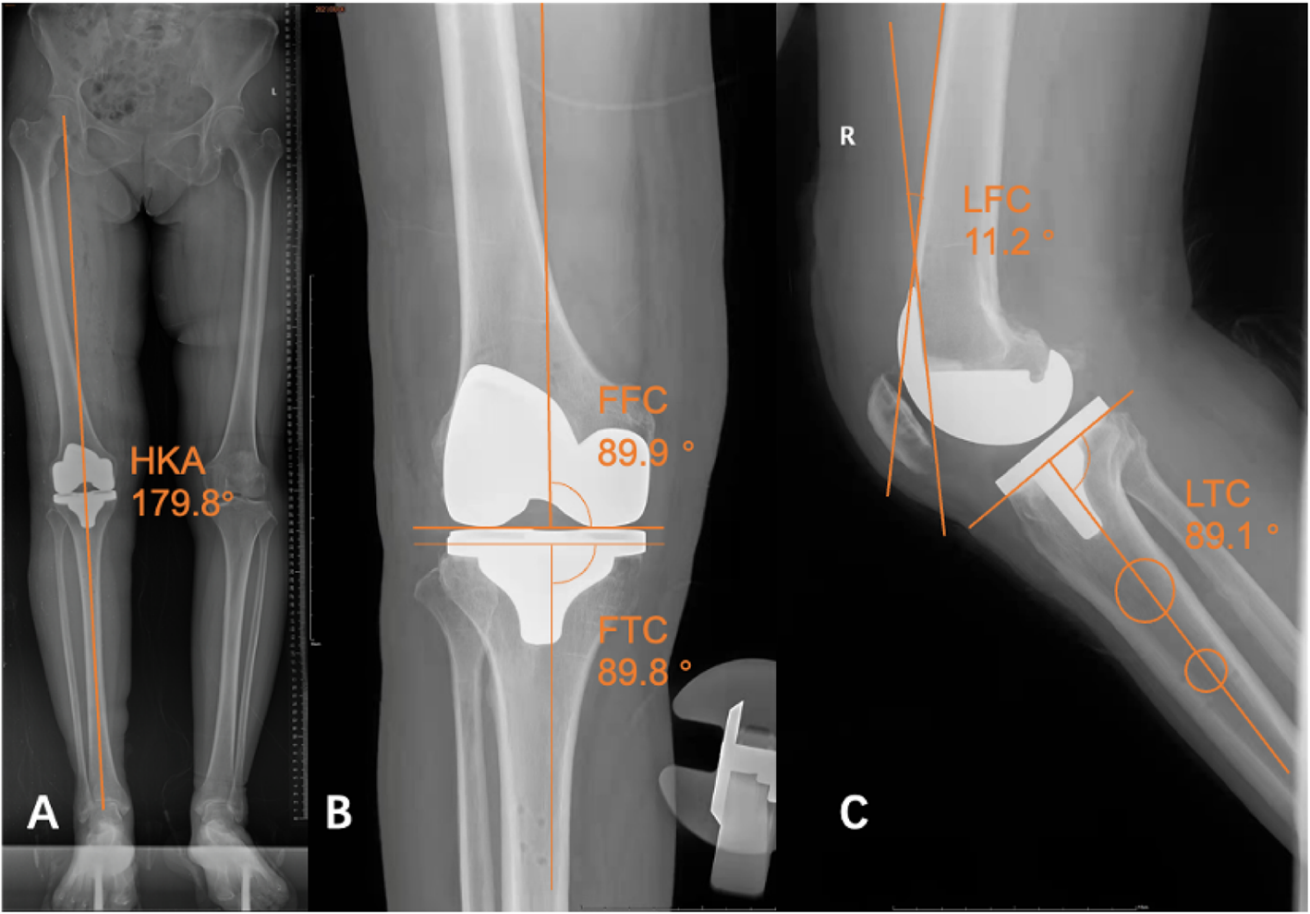
Measurement of evaluated angles. **A** Measurement of the postoperative HKA angle. **B** Measurement of the FFC angle and FTC angle. **C** Measurement of the LFC and LTC angles

Weight-bearing full-length X-ray of the lower extremity was taken 2 days after surgery to measure the following: ①frontal femoral component (FFC, optimum 90°) measured by front and side X-ray of the knee joint; ②frontal tibial component (FTC, optimum 90°); ③ lateral femoral component (LFC, optimum 11°, based on the characteristics of the prosthesis used in this trial and after consultation with clinicians); ④lateral tibial component (LTC, The optimum value for the CM-TKA group is 87°, with an optimum value of 89° for the MA-TKA group); and ⑤ HKA (hip-knee-ankle, optimum 180°) [3] (Fig. 1). Outliers were calculated as the difference between the above angles and the optimum value of ≥3 °[17].

Each angle measurement was performed independently by 2 orthopaedic surgeons using Digimizer (V 5.4.3) software, and the final results were averaged between the two. If the difference between the two measurements was too large (≥0.5°), then a third senior orthopaedic surgeon measured the angle and provided the final result, then the average of the two similar measurements among the three was taken.

### Statistical methods

SPSS 26.0 statistical software (IBM, USA) was used to statistically analyse the relevant data. The Kolmogorov–Smirnov test was used to assess whether the variables followed a normal distribution. Measures that were tested to conform to a normal or approximately normal distribution were expressed as the mean ± standard deviation (SD), and those that did not conform to a normal distribution were expressed as the median (Q_1_, Q_3_). According to the normality test, two independent samples t tests or Mann–Whitney U tests were used for continuous variables. Count data such as patient gender distribution and affected side distribution were described as n (%), and differences were compared using the chi-square test or Fisher’s exact test. Differences were considered statistically significant if the *P* value was < 0.05.

## Results

The procedures were successfully completed in both groups; the tourniquet use time was longer than in the MA-TKA group but the difference was not significant (77.3 ± 15.8 vs. 66.1 ± 17.7 mins, *p* = 0.078). The calculated EBL was significantly lower in the MA-TKA group than in the CM-TKA group (496.9 ± 257.8 vs. 773.0 ± 301.3 ml, *p* < 0.001). At the 6-week postoperative outpatient follow-up, there were no significant differences in ROM, KSS or WOMAC scores between the two groups (*p* > 0.05) (Table 2).

**Table 2:** Surgical data and clinical outcomes in the CM-TKA and MA-TKA groups

Parameter	MA- TKA (*n* = 34)	CM- TKA (*n* = 31)	*P* value
**Preoperative**			
ROM(°)	100.4 ± 23.2	93.4 ± 16.3	0.165
KSS	36.6 ± 12.1	41.9 ± 10.5	0.068
WOMAC	87.1 ± 7.2	87.4 ± 5.0	0.846
**Operative**			
Tourniquet duration (min)	77.3 ± 15.8	66.1 ± 17.7	0.078
Estimated blood loss (ml)	496.9 ± 257.8	773.0 ± 301.3	0.001
**Postoperative**			
Week 6 ROM(°)	119.1 ± 2.9	118.4 ± 5.6	0.523
Week 6 KSS	85.5 ± 3.0	84.1 ± 3.3	0.078
Week 6 WOMAC	15.9 ± 2.6	16.0 ± 2.9	0.827

*MA-TKA* MAKO-robotic assisted total knee arthroplasty, *CM-TKA* conventional manual total knee arthroplasty, *ROM* range of motion, *KSS* American Knee Society Score, *WOMAC* Western Ontario McMaster Universities Osteoarthritis Index, *EBL* estimated blood loss

There was no significant difference in preoperative serum inflammatory markers between the two groups (Table 3); there was no significant difference in white blood cell count, neutrophil-to-lymphocyte ratio NLR, CRP, ESR or creatine kinase on the first postoperative day between the two groups (p>0.05); but IL-6 levels were significantly lower in the MA-TKA group (11.4 (5.2, 21.0) vs. 24.6 (8.6, 124), *p* = 0.031). CRP, ESR, IL-6, and CK were significantly higher in the CM-TKA group than in the MA-TKA group on postoperative Day 3 (72 h) (*p* < 0.05).

**Table 3:** The lab test results of CM- TKA and MA- TKA groups

Parameter	MA-TKA (*n* = 34)	CM-TKA (*n* = 31)	*P* value
**Preoperative**			
White cell count	7.4 ± 3.4	6.7 ± 1.9	0.306
NLR	2.7 ± 2.5	2.5 ± 1.2	0.641
CRP	0.1 (0.08, 0.22)	0.1 (0.05, 0.14)	0.647
ESR	13.2 ± 11.3	12.1 ± 7.0	0.64
IL-6	5.9 ± 11.9	5.0 ± 3.9	0.697
CK	85.7 ± 66.7	79.5 ± 38.3	0.654
**Day 1**			
White cell count	12.8 ± 4.4	11.1 ± 2.8	0.06
NLR	13.2 ± 7.6	10.4 ± 4.8	0.074
CRP	0.45 (0.1, 1.1)	0.77 (0.3, 1.7)	0.141
ESR	11.6 ± 8.2	13.5 ± 9.0	0.371
IL-6	11.4 (5.2, 21.0)	24.6 (8.6, 124.0)	0.031
CK	116.5 (84.7, 171.7)	122 (86.3, 170.8)	0.844
**Day 3**			
White cell count	9.4 ± 3.8	8.8 ± 2.3	0.433
NLR	5.0 ± 2.8	5.8 ± 4.0	0.391
CRP	2.0 ± 2.7	5.3 ± 3.2	0.001
ESR	17.3 ± 16.3	27.9 ± 13.3	0.006
IL-6	5.1 (2.0, 22.4)	24.0 (6.0, 36.1)	0.023
CK	75.7 (57.1, 130.0)	106.1 (78.2, 175.4)	0.046

*MA-TKA* MAKO-robotic assisted total knee arthroplasty, *CM-TKA* conventional manual total knee arthroplasty, *NLR* Neutrophil-to-lymphocyte ratio, *CRP* C-reactive protein, *ESR* erythrocyte sedimentation rate, *IL-6* interleukin-6, *CK* Creatine Kinase

There was no difference in preoperative HKA between the two groups; however, imaging performed on the second postoperative day showed that HKA was significantly better in the MA-TKA group than in the conventional TKA group (178.7 ± 1.3° vs. 177.8 ± 2.2°, *p* = 0.041), and only 2 cases were outliers (Table 4), which was significantly better than the 12 outlier cases in the CM-TKA group (5.9% vs. 38.7%, *p* < 0.001). In the comparison of FFC angles, the RA-TKA group was significantly closer to the expected value of 90° (90.9 (90.5, 92.3) vs. 92.4 (91.3, 93.7), *p* = 0.031), but the probability of outliers was not significantly different between the two groups (17.6% vs. 32.3%, *p* = 0.172). No significant differences were found between the two groups in the comparison among the remaining angles in terms of values or outliers (*p* > 0.05).

**Table 4:** Comparison of imaging findings between the two groups postoperatively

Parameter	MA-TKA (*n* = 34)	CM-TKA (*n* = 31)	*P* value
HKA (°)	178.7 ± 1.3	177.8 ± 2.2	0.041
Outlier (≥3°)	2 (5.9%)	12 (38.7%)	0.001
FFC (°)	90.9 (90.5, 92.3)	92.4 (91.3, 93.7)	0.031
Outlier (≥3°)	6 (17.6%)	10 (32.3%)	0.172
FTC (°)	89.6 ± 1.8	89.0 ± 2.2	0.233
Outlier (≥3°)	4 (11.7%)	4 (12.9%)	0.889
LFC (°)	11.0 ± 3.1	10.4 ± 3.2	0.462
Outlier (≥3°)	11 (32.3%)	11 (35.5%)	0.410
LTC (Diff. from Optimum,°)	0.9 (0.3, 1.0)	1.4 (0.6, 2.3)	0.019
Outlier (≥3°)	1 (2.9%)	1 (3.2%)	0.914

*MA-TKA* MAKO-robotic assisted total knee arthroplasty, *CM-TKA* conventional manual total knee arthroplasty, *HKA* hip-knee-ankle, *FFC* frontal femoral component, *FTC* frontal tibial component, *LFC* lateral femoral component, *LTC* lateral tibial component

## Discussion

### Operation and early clinical results

A review by Vermue, H. et al. mentioned that the learning curve of RA-TKA includes 6-20 procedures [18]. However, the operators performed more than 20 RA-TKA procedures prior to the first procedure in this study and successfully passed through the learning curve. Studies have shown that a shorter duration of tourniquet use leads to faster recovery and quick pain relief in the early recovery period after TKA [19]. Although there was no significant difference, the MA-TKA group in this study had an approximately 11-minute longer tourniquet application time, which may be related to the fact that we placed the fixation staple first and then inflated the tourniquet while cutting the skin. The conventional perception is that the RA-TKA extended tourniquet time is mostly used for repeat registration and the alignment of femoral and tibial anatomical markers, but in routine practice, we have found that spending the time to get past the initial learning curve substantially reduces the incidence of repeat registration. We believe that the extended tourniquet application time of MA-TKA during this study may be related to repeated prebalancing tests performed when pre-osteotomy is inadequately revealed and the additional time associated with performing manual pre-osteotomy in patients with heavy deformities and extra bone fragments.

As mentioned in some of the previous studies, the use of RA-TKA compared to CM-TKA showed lower EBL [20–22]. In this study, EBL was calculated by the haemoglobin balance method at 72 hours postoperatively in both groups, and the results suggested that it was significantly lower in the MA-TKA group. The use of an intramedullary positioning system to disrupt the medullary cavity during CM-TKA is thought to result in significant blood loss during routine total knee arthroplasty and may also result in extravasation of blood, which leads to occult blood loss [20]. MA-TKA, on the other hand, prevents damage to the femoral medullary cavity and thus reduces blood loss by locating important bony landmarks prior to osteotomy, thus determining the osteotomy position required for implant placement based on preoperative CT and localization registration.

In this study, ROM, KSS and WOMAC scores were measured at 6 weeks postoperatively in both groups, and there was no significant difference between the two groups. There is controversy as to whether the use of RA-TKA results in better postoperative clinical scores, and a study by Kayani, B et al. found that RA-TKA reduced pain, improved early functional recovery, and reduced time to discharge [23]. Marchand, K. B. et al. also concluded, after a 2-year follow-up on robotic TKA and conventional TKA, that patients who underwent RA-TKA had improved outcomes 2 years after the surgery compared with patients who underwent conventional TKA [24]. However, in contrast, Singh, V. et al. concluded that although the use of robot-assisted technology may help surgeons, its use has not translated into better short-term outcomes at the 1-year follow-up [25]. Different ways of evaluating surgical outcomes can suggest different results, and we will continue to follow up to see if this improves clinical outcomes.

### MA-TKA reduces the postoperative inflammatory response in the short term

In this study, MA-TKA patients had significantly lower levels of IL-6 on the first postoperative day, and this difference in the inflammatory response became more pronounced on the third postoperative day, with patients in the MA-TKA group having significantly lower levels of CRP, ESR, and IL-6 than those in the CM-TKA group. Kayani, B. et al. found that robotic TKA was associated with a transient reduction in the inflammatory response in the early postoperative period (Day 7) but found no difference in the systemic inflammatory response in the immediate (< 48 hours) or late postoperative period (Day 28) compared with CM-TKA [7]. This study presents, for the first time, the advantages of RA-TKA in postoperative recovery through a comparison of blood markers. However, the study recorded no indicators in the time between postoperative Days 2 and 7, even though the peak changes in inflammatory indicators such as IL-6 and CRP occurred in this period. Therefore, we believe that the difference between MA-TKA and CM-TKA partial inflammatory indices at 3 days postoperatively (72 h) may be more representative. This study illustrates that the difference in inflammatory indicators between the two groups of patients at 3 days postoperatively is significant. In conjunction with the study by Kayani, B. et al., the difference in the inflammatory response after RA-TKA versus CM-TKA was observed at least 3 to 7 days postoperatively. We speculate that this is related to the fact that MA-TKA did not use intramedullary localization during femoral osteotomy and that the use of robotic arm osteotomy prevents soft tissue injury and reduces ligamentous release [26]. Although serum inflammatory markers are an indirect representation of surgical outcomes, they are very objective. These differences may be reflected in incision healing, lower extremity blood clots, and surgical site pain.

CK, as a muscle damage marker, is mainly found in the cytoplasm and mitochondria and is an important kinase directly related to intracellular energy functioning, muscle contraction, and ATP regeneration. It has been shown that the prolonged use tourniquets significantly increases CK levels due to the increased duration of ischaemia–reperfusion injury [27]. An increase in CK may adversely affect postoperative functional outcomes [28]. In contrast, the tourniquet use time in the MA-TKA group in this study was longer but was associated with lower CK levels at 72 h postoperatively. Although the CK was in the normal range in both groups, the MA-TKA group had a longer tourniquet time but showed a slightly greater magnitude of CK change, which we believe is because MA-TKA did not require complete exposure during the osteotomy because the robotic arm limited the osteotomy range. Reduced intensity and persistent tension at the incision site resulted in lower CK levels in the MA-TKA group.

### RA-TKA has a more ideal placement of the prosthesis

RA-TKA has been reported several times in recent years to improve the postoperative imaging prosthesis position, and Batailler, C. et al. analysed 26 studies on MA-TKA and concluded that the CT-based robot-assisted system reduced postoperative pain and improved prosthesis positioning after TKA compared to CM-TKA [29]. Elliott, J. et al. conducted a retrospective analysis of 39 published RA-TKA-related studies, which added to the evidence supporting the use of robot assistance in TKA to improve accuracy and reproducibility [30].

As with sex, people of different ethnicities can have different anatomical characteristics. One study found that, compared with the white population, Chinese females and males have a more substantially valgus anatomic axis, females have more valgus condylar angles (angle between the mechanical or anatomic axis line of the femur and a line tangent to the femoral condyles), and males have more valgus condylar-plateau angles (angle between the condylar angle and tibial plateau angle) [31]. Therefore, it is still unknown whether this robot-assisted system will help the surgeon provide better prosthesis placement in Chinese patients undergoing TKA. The radiographic results reported on the second postoperative day in this study also suggested that MA-TKA was more advantageous in placing the postoperative prosthesis in the precise angle position. The MA-TKA group was significantly less likely to have abnormal postoperative HKA values and was significantly closer to the preoperative expected 180° lower extremity force line than the CM-TKA group. After measuring the femoral component angle in the coronal plane, we found that although there was no significant difference in the probability of occurrence of abnormal values, the MA-TKA group was significantly closer to the expected values. Theoretically, better lower extremity force lines and angulation of the prosthesis position would reduce prosthesis wear, but Johannes Cip et al. found no differences in long-term survival, implant accuracy, clinical outcomes, or score outcomes between RA-TKA and CM-TKA after 12 years of follow-up [4]. This is related to the technology used in different generations of robots, and the advantages of the higher accuracy of RA-TKA need to be discovered in a large sample of long-term follow-up studies.

There are some limitations in this study. First, this study is a retrospective study, but the preoperative clinical characteristics of our two groups of patients were not significantly different and were comparable, which reduced the extent of differences between the groups. Second, the follow-up period was short, but a large sample and long-term follow-up are needed to verify the conclusions obtained. In the analysis of inflammatory indices, due to hospital conditions, patients were usually discharged 3 days after surgery if they recovered without complications, so follow-up was not conducted. Finally, radiographic results were evaluated using X-rays at 2 days postoperatively, and patients often did not fully extend their lower extremities at that time, which lead to difficulties in determining the results. However, we used the same posture and angle during the examination to eliminate bias.

## Conclusion

In this study, we found that MA-TKA patients had less EBL and a milder systemic inflammatory response in the early postoperative period. In Chinese patients with OA, MA-TKA showed a more precise prosthesis position and better lower extremity force lines than CM-TKA. However, even when the operator passed through the learning curve, the tourniquet time was still prolonged, and MA-TKA did not show better functional or pain scores in the short-term follow-up. Further follow-up is needed for medium- and long-term outcomes.

## Data Availability

Data are available on request from the authors.

## Abbreviations

CM-TKA: Conventional manual total knee arthroplasty
RA-TKA: MAKO-robotic assisted total knee arthroplasty
EBL: Estimated blood loss
ROM: Range of motion
KSS: American Knee Society Score
WOMAC: Western Ontario and McMaster Universities Osteoarthritis Index
CRP: C-reactive protein
ESR: Erythrocyte sedimentation rate
IL-6: Interleukin-6
CK: Creatine kinase
NLR: Neutrophil-to-lymphocyte ratio
HKA: Hip-knee-ankle angle
FFC: Frontal femoral component
FTC: Frontal tibial component
LFC: Lateral femoral component
LTC: Lateral tibial component
